# DIRAS2 Is a Prognostic Biomarker and Linked With Immune Infiltrates in Melanoma

**DOI:** 10.3389/fonc.2022.799185

**Published:** 2022-05-16

**Authors:** Wenli Xue, Hongbo Zhu, Hongye Liu, Hongxia He

**Affiliations:** ^1^ Department of Dermatology, The First Hospital of Shanxi Medical University, Tai Yuan City, China; ^2^ Department of Medical Oncology, The First Affiliated Hospital, Hengyang Medical School, University of South China, Hengyang, China

**Keywords:** DIRAS2, skin cutaneous melanoma, immune infiltrates, Wnt/β-catenin signaling pathway, prognosis

## Abstract

**Background:**

Skin cutaneous melanoma (SKCM) is a highly malignant skin tumor. DIRAS2 is considered to be a tumor suppressor gene; however, its function in SKCM has not been explored.

**Methods:**

The Gene Expression Profiling Interactive Analysis (GEPIA) was implemented to investigate the expression of DIRAS2 in SKCM, and plot the survival curve to determine the effect of DIRAS2 on the survival rates of SKCM patients. Then, the correlation between DIRAS2 and tumor immune infiltration was also discussed, and the expression of DIRAS2 and immune infiltration level in SKCM immune cells was determined using TIMER. The top 100 genes most associated with DIRAS2 expression were used for functional enrichment analysis. In order to confirm the anti-cancer effects of DIRAS2 in SKCM in the data analysis, *in vitro* assays as well as *in vivo* studies of DIRAS2 on SKCM tumor cell proliferation, migration, invasion, and metastasis were conducted. Western blot and immunofluorescence assay were employed to study the relationship between DIRAS2 and Wnt/β-catenin signaling pathway in SKCM.

**Results:**

DIRAS2 expression was shown to be significantly correlated with tumor grade using univariate logistic regression analysis. DIRAS2 was found to be an independent prognostic factor for SKCM in multivariate analysis. Of note, DIRAS2 expression levels were positively correlated with the infiltration levels of B cells, CD4+ T cells, and CD8+ T cells in SKCM. The infiltration of B cells, CD4+ T cells, and CD8+ T cells was positively correlated with the cumulative survival rate of SKCM patients. *In vitro* experiments suggested that proliferation, migration, invasion, and metastasis of SKCM tumor cells were distinctly enhanced after DIRAS2 knockdown. Furthermore, DIRAS2 depletion promoted melanoma growth and metastasis *in vivo*. As for the mechanism, silencing DIRAS2 can activate the signal transduction of the Wnt/β-catenin signaling pathway.

**Conclusion:**

DIRAS2 functions as a tumor suppressor gene in cases of SKCM by inhibiting the Wnt/β-catenin signaling. It is also associated with immune infiltration in SKCM.

## Introduction

Skin cutaneous melanoma is a type of skin tumor that is highly malignant. Because of its aggressive nature and high mortality, it is the most dangerous form of skin cancer (1). The prognosis of melanoma is closely related to the tumor stage because of its rapid progression, easy metastasis, and high mortality rates (1). Melanoma stages are classified as I-IV, and as the tumor metastasizes, either locally or distantly, the stage progresses beyond stage III. The overall survival rate for stage I-II patients is between 75 and 98 percent. Unfortunately, however, one-third of these victims develop metastatic melanomas. The 10-year overall survival rate for patients in stages IIIA-D was significantly lower, ranging from 24% to 88% (2). In the early stages of the disease, surgical treatment is usually curative (3, 4). However, once melanoma metastasizes, its life-threatening risk greatly increases (5). Despite significant advances in targeted therapies and novel immunotherapies (6, 7), the efficacy of all treatments is greatly compromised as compared to active surgical treatment, which is very effective in the early stages of the disease. Thus, there is a need to identify biomarkers that are related to tumors in conjunction with stages that affect prognosis (8–10).

Members of the GTP-binding Ras-like protein (DIRAS) family may function as tumor suppressors in a range of cancers, according to several reports. The loss or decrease of DIRAS protein expression is critical in the development of cancer (11). The DIRAS family includes three functional genes (*DIRAS1*, *DIRAS2*, and *DIRAS3*) (12). Sutton Mn et al. showed that the expression of members of the DIRAS family was diminished in mouse ovarian cancer cell lines. Experiments *in vitro* confirmed that overexpression of DIRAS1 and DIRAS2 reduced the growth of ovarian cancer cells (13). DIRAS2 regulated the phosphorylation of downstream effector proteins and activated the RAS/mitogen-activated protein kinase (MAPK) signaling pathway, according to another research by Rao H et al. (14). However, the role of DIRAS2 in SKCM has not been investigated (i.e., whether it is a tumor suppressor gene or an oncogene).

In this study, Rand Gene Expression Profiling Interactive Analysis was used to identify correlations between DIRAS2 and SKCM utilizing data from The Cancer Genome Atlas (TCGA) (GEPIA). Then, new meta-gene approaches, CIBERSTE and Tumor Immune Estimation Resource (TIMER) were implemented to measure, in different tumor microenvironments, the density of numerous tumor-infiltrating immune cells (TIICs). Finally, in order to detect whether DIRAS2 affected SKCM growth, proliferation, and/or migration, we carried out a series of cytological experiments that involved down-regulation of DIRAS2 and deeply studied tumor cell functions. Our study confirmed the tumor-suppressing properties of DIRAS2; it is indeed a tumor suppressor gene in SKCM.

## Materials and Methods

### Data Acquisition

A dataset of SKCM patients from the public TCGA was collected, which included gene expression profiles and clinical information. This collection includes 558 normal tissues and 461 tumor tissues. Following the deletion of the missing clinical data sets, RNA sequencing data was converted into microarray-like values by transforming count data. Tumor tissues were next divided into two groups based on DIRAS2 expression, and the effects of DIRAS2 expression on the immune microenvironment of SKCM patients were studied. For verification, the GEO dataset GSE15605 was obtained.

### Expression and Survival Analysis by GEPIA

GEPIA (http://gepia.cancer-pku.cn/index.html) is used for the interactive analysis of gene expression profiles. It can be applied to single-gene analysis, multigene analysis, and tumor type analysis. GEPIA was used in this research to validate DIRAS2 expression levels in SKCM and to analyze the effects of DIRAS2 expression on SKCM patient survival rates. Following that, pathological stages were used as variables to examine staging diagrams, and we compared DIRAS2 expression at different pathological stages of SKCM. Additionally, boxplots with disease states (tumor or normal) as variables were plotted to calculate differential DIRAS2 expression.

### Immunoinfiltration Analysis by TIMER and CIBERSORT

TIMER was used to systematically analyze various types of tumor immune information (https://cistrome.shinyapps.io/timer/). The database contained 10,897 samples from 32 cancers from TCGA, and the correlation between malignancies and immune cell infiltration was systematically examined. To verify the expression and prognosis of this gene in SKCM, “Gene Symbol: DIRAS2” and “Cancer Type: SKCM” were entered into the “Gene” and “Survival” modules. The infiltration of immune cells, including B cells, CD4+ T cells, CD8+ T cells, neutrophils, macrophages, and dendritic cells, in SKCM patients’ tissues, as well as the correlation between the prognosis and DIRAS2, were analyzed.

CIBERSORT (15) (http://cibersort.stanford.edu/”http://cibersort.stanford.edu/) is the most widely-used immune cell infiltrating evaluation analysis tool. CIBERSORT enables the reliable and replicable analysis of cellular heterogeneity in genomic data sets (16), including fresh/frozen tissue and fixed clinical specimens. Samples showed that the immune cell subsets computed by CIBERSORT were accurate and could be compared between different immune cell types and datasets. We obtained RNA-Seq data from 461 SKCM patients’ tumor tissue samples from the TCGA database and converted them into 22 different immune cell compositions using the CIBERSORT website. To study the effects of differential DIRAS2 expression levels on the immune microenvironment, we divided them into two groups based on the median. We investigated the differences in the abundance of immune cell infiltration between high and low DIRAS2 expression groups.

### Statistical Analysis by R-3.5.3

To assess the relationship between clinical information and DIRAS2 expression, we used logistic regression on TCGA data acquired using R3.5.3. A multivariate Cox analysis was applied to analyze DIRAS2 expression, as well as the effects of age on survival rate. P < 0.05 was the critical value. To study correlations between 22 immune cells, a correlation heat map (i.e., a correlation chart between every pair of two immune cells in the sample) was constructed.

### Gene Enrichment Analysis

The top 100 genes with a significant correlation with diras2 expression in SKCM were obtained from GEPIA. Gene ontology (GO) and Kyoto Encyclopedia of Genes and Genome (KEGG)analyses were conducted on the top 100 genes with the cluster Profiler, enrichplot, and ggplot2 packages.

### Cell Culture and Tissue

The SKCM cell lines A375, B16-F10, and SK-MEL-1 as well as normal HaCaT were derived from the Shanghai cell bank of the Chinese Academy of Sciences, subcultured and preserved in liquid nitrogen. All cell lines were cultured in a 37°C, 5% carbon dioxide incubator using 10% fetal bovine serum and the RPMI-1640 medium (which contained 1% penicillin and streptomycin mixture).

Three SKCM samples came from Shanxi Medical University’s First Hospital, while three healthy skin samples came from Hengyang Medical School’s First Affiliated Hospital. No patients had previously received treatment, and all patients provided informed consent. All tissue samples were extracted with protein and then subjected to western blot.

### Real-Time Fluorescence Quantitative PCR (qPCR)

Tranzol up was used to extract the total RNA from the cells, and a microplate reader was used to evaluate the concentration and purity of the total RNA sample, which was used for reverse transcription of the total RNA to generate cDNA. After the cDNA was diluted, 1 μL was utilized as a template for real-time fluorescence quantitative PCR. Primer sequences of target gene DIRAS2 and internal reference gene GAPDH were:

DIRAS2: Forward Primer: 5’-GCAGTGACCCTGGAGAGG-3’;
Reverse Primer: 5’-TCAAGTCCTCGTGCACAATC-3’;
GAPDH: Forward Primer: 5’-CAGGAGGCATTGCTGATGAT-3’;
Reverse Primer: 5’-GAAGGCTGGGGCTCATTT-3’.

### CCK-8 Method

The cells were collected during the logarithmic growth period, counted them, diluted the cell suspension to an appropriate concentration, inoculated them into96 well plates, and set 3 multiple wells in each group at 0, 24, 48, and 72 hours. At each time point, the 10μL CCK-8 solution was added. The 96 well plates were stored in the incubator and continued the culture for 4 hours. A microplate reader was used to measure absorbance at 450 nm at the end of the culture.

### Plate Clone Formation Experiment

SKCM cell lines in the logarithmic growth stage were digested, washed with PBS once, resuspended with fresh medium, and counted for standby. The cell suspension was diluted using gradient multiple dilutions. Cells from each group were inoculated into six-well plates. Cells were cultured for 2 weeks in a 37°C constant temperature incubator (with 5% CO_2_ and saturated humidity). The culture was terminated when the cells developed visible cell clones, which were dyed with crystal violet. The number of clones was directly counted and calculated the clone formation rate = (number of clones/number of inoculated cells) ×100%.

### Western Blot

The proteins from the cell samples were extracted and the BCA method was used to determine the protein concentration. After adding loading buffer, the mixture was heated in a boiling water bath for 10 minutes before being cooled to room temperature. DIRAS2 (Abcam, 1:1000), GAPDH (Abcam, 1:10000), Wnt-1(Abcam, 1:1000), β-catenin (Abcam, 1:1000), and c-MYC (Abcam, 1:1000) protein expression were detected by polyacrylamide gel electrophoresis assay.

### Immunofluorescence Staining

The sections were fixed and then blocked with 10% goat serum and 0.03% Triton X-100 blocking solution for 2h at room temperature, followed by the addition of rabbit monoclonal myc (Abcam, 1:500) primary antibody at 4°C overnight. 1:1000 dilution of fluorescent secondary antibody were added and incubated in the incubator at room temperature for 2h and then washed. The slides were sealed with an anti-fluorescence quenching agent and stored at 4°C, and then the slides were observed under a laser confocal microscope.

### Immunohistochemical Assay

Patient tissues were fixed in 4% formaldehyde and then treated by embedding. After sectioning, washing, antibody incubation, dehydration and sealing with neutral gum, the tissues were evaluated by an experienced physician in the hospital pathology department in a double-blind method. Five dense areas of positive cells were selected under a 100x field of view, and the average number of positive cells in the five areas was taken as the result of the section. 0 for no positive cells or a positive rate of <10%, 1 for 10% to <25%, 2 for 25% to <50%, 3 for 50% to <75% and 4 for ≥75%. Depth of staining: no staining 0, light staining 1, moderate staining 2, deep staining 3. The final immunohistochemical score for protein in tissue is obtained by multiplying the positive cell score with the staining score.

### Animal Studies

A375 cells were injected into the same subcutaneous location on the back of nude mice at a rate of 5x10^6^ cells per tumor. Every week, the tumor size was measured, and after 4 weeks, the nude mice were executed and the tumors were removed. NOD/SCID mice were used to create an animal model of melanoma metastasis. Briefly, 5x10^5^ A375 cells were injected into the mice into the tail vein once a day for 3 days. Finally, the mice were euthanized, and H&E staining was used to identify the lung metastases. All methods were authorized by the Animal Care and Use Committees of Shanxi Medical University’s First Hospital and Hengyang Medical School.

### Statistical Method

GraphPad 5.0 software was used to process the data presented above. The standard deviation was used to analyze the mean of more than two groups using analysis of variance (ANOVA) tests. P < 0.05 implied statistical significance.

## Results

### Differential Expression and Survival Analysis of DIRAS2 in SKCM

**Figure 1:**
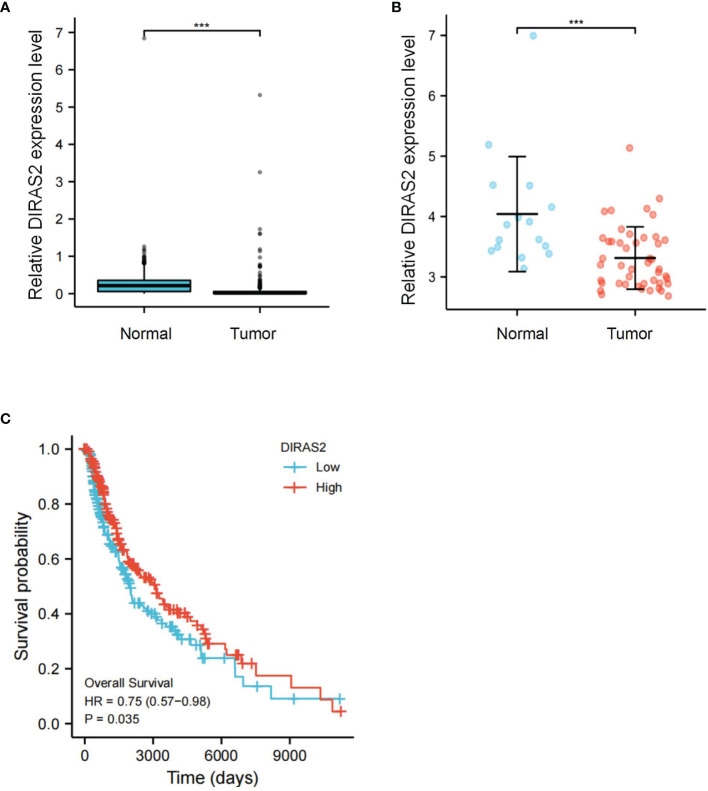
Differential expression and survival analysis of DIRAS2 in SKCM. **(A)** Differential expression of DIRAS2 in different states (Tumor or Normal) of TCGA dataset. **(B)** Differential expression of DIRAS2 in different GEO dataset states (Tumor or Normal) (GSE15605). **(C)** GEPIA survival curves of differential DIRAS2 expression. ***p < 0.001.

Our cohort from the TCGA database represented SKCM patients with pathologic stages of I and III, aged both < =60 and > 60. Seventy-seven patients (18.7%) were at pathologic stage I, and 171 patients (41.5%) were at pathologic stage III. Boxplot expression was done by GEPIA, as shown in **Figure 1A**, and revealed decreased expression of DIRAS2 in SKCM compared to normal skin (P < 0.001). In GEO, a similar result can be verified (**Figure 1B**). Overall survival analysis indicated that low expression levels of DIRAS2 might predict worse prognoses in patients with SKCM (**Figure 1C**). Additionally, univariate Cox regression analysis manifested that DIRAS2 status (P = 0.024), age (<=60 and > 60, P <0.001), and pathological stage (P < 0.001) were significantly correlated with SKCM overall survival (**Table 1**). Pathologic stage (P = 0.001) and DIRAS2 expression (P = 0.021) were shown to be independent prognostic factors in multivariate analysis.

**Table 1:** Multivariate Cox analysis of DIRAS2 expression and other clinical-pathological factors.

Characteristics	Total(N)	Univariate analysis		Multivariate analysis	
		Hazard ratio (95% CI)	P value	Hazard ratio (95% CI)	P value
DIRAS2 (High vs. Low)	456	0.735 (0.562-0.961)	0.024	0.653 (0.455-0.937)	0.021
Pathologic stage (Stage I vs. Stage III)	247	0.489 (0.328-0.730)	<0.001	0.491 (0.329-0.734)	<0.001
Age (>60 vs. <=60)	456	1.656 (1.251-2.192)	<0.001	1.358 (0.926-1.991)	0.117

### DIRAS2 Expression Correlates With Immune Infiltration

**Figure 2:**
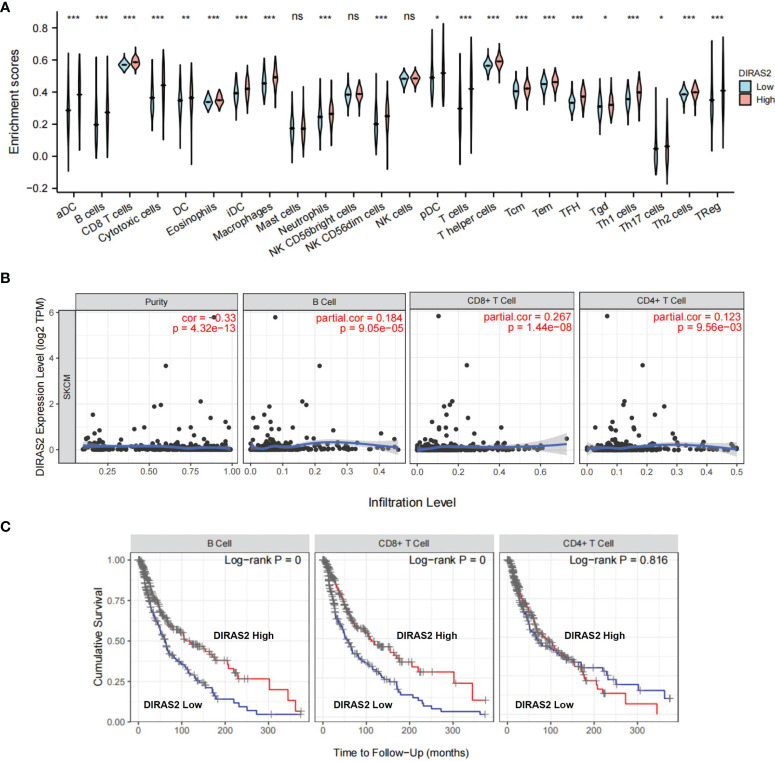
Immune cells are associated with SKCM survival rates. **(A)** The proportion of 22 immune cell subpopulations. aDC [activated DC]; B cells; CD8 T cells; Cytotoxic cells; DC; Eosinophils; iDC [immature DC]; Macrophages; Mast cells; Neutrophils; NK CD56bright cells; NK CD56dim cells; NK cells; pDC [Plasmacytoid DC]; T cells; T helper cells; Tcm [T central memory]; Tem [T effector memory]; Tfh [T follicular helper]; Tgd [T gamma delta]; Th1 cells; Th17 cells; Th2 cells; Treg. **(B)**DIRAS2 expression levels were significantly positively correlated with infiltrating levels of B cells and T cells in SKCM. **(C)** In SKCM, cumulative survival was related to B and T cells. *p < 0.05; **p < 0.01; ***p < 0.001; ns, no significance.

Tumor-infiltrating lymphocytes are known to independently predict sentinel lymph node status and overall survival rates for cancer patients (17). This information prompted us to explore the relationship between DIRAS2 expression and immune infiltration levels in SKCM, as well as other specific correlations. TCGA-downloaded data included 236 DIRAS2 high expression samples and 235 low expression samples. The gene expression profiles of the downloaded samples were first studied using CIBERSORT to evaluate the density of 22 immune cells. It was found that DC, B cells, CD8 T cells, cytotoxic cells, eosinophils, iDC, Macrophages, Neutrophils, NK CD56dim cells, T cells, T helper cells, Tcm, Tem, TFH, Th1 cells, Th2 cells, TReg were the main immune cells affected by DIRAS2 expression (**Figure 2A**). The number of immune cell infiltrates in the DIRAS2 high expression group was significantly higher than in the DIRAS2 low expression group (P < 0.0001).

In SKCM patients, DIRAS2 expression levels were positively correlated (**Figure 2B**) with infiltration levels of B cells (r = 0.184, P = 9.05, e-05), CD4 + T cells (r = 0.123, P = 9.56, e-03), CD8 + T cells (r = 0.267, P = 1.44, e-08). Furthermore, our results highlight the importance of DIRAS2 in SKCM immune infiltration, since B cells, CD8 + T cells, and CD4 + T cells were related to SKCM survival rates over time (**Figure 2C**).

### Enriched Pathway Analyses of DIRAS2 in SKCM

**Figure 3:**
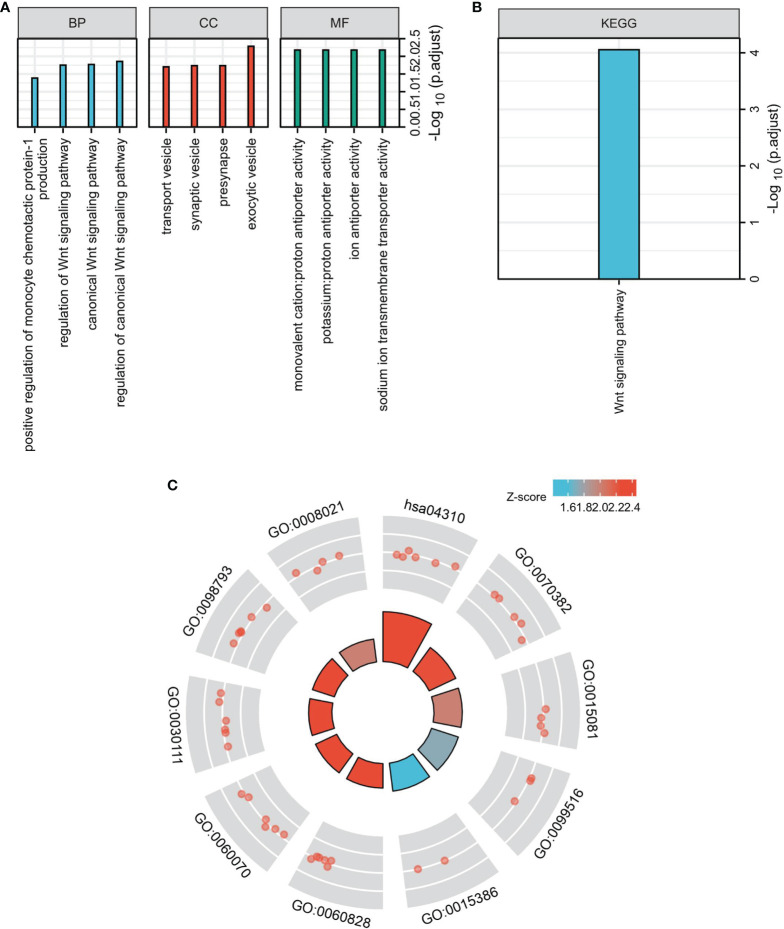
Functional enrichment analysis to clarify DIRAS2 function. **(A)** Histogram of signaling pathways affected by DIRAS2. Enrichment analysis based on GO annotation classification. **(B)** Analysis of enrichment based on the KEGG pathway’s annotated classification. **(C)** Bubble diagram of signaling pathways affected by DIRAS2, combining GO annotation classification and KEGG pathway annotation classification.

The top 100 genes in the GEPIA database were selected, that have the highest relevance to DIRAS2 expression in SKCM to explore the pathways that DIRAS2 may be involved in modulating tumor progression in SKCM (**Supplementary Table 1**). Furthermore, clustering analysis of these 100 genes was performed, including GO analysis with KEGG analysis. By GO enrichment analysis, the cellular component (CC), biological process (BP), and molecular function (MF) categories were screened based on the criteria of p-value < 0.01 and FDR < 0.05. DIRAS2 was associated with BP, including regulation of the canonical Wnt signaling pathway, and CC, which included exocytic vesicle, presynapse, and synaptic vesicle. The sodium ion transmembrane transporter activity, ion antiporter activity, and proton antiporter activity were all increased in MF (**Figure 3A**). KEGG pathway analysis confirmed the Wnt signaling pathway as an enrichment pathway (**Figures 3B, C** and **Table 2**).

**Table 2:** Enriched Pathway Analyses of DIRAS2 in SKCM.

Ontology	ID	Description	GeneRatio	BgRatio	p-value	p.adjust	q-value
BP	GO:0060828	regulation of canonical Wnt signaling pathway	6/34	286/18670	1.15e-05	0.014	0.011
BP	GO:0060070	canonical Wnt signaling pathway	6/34	335/18670	2.81e-05	0.017	0.013
BP	GO:0030111	regulation of Wnt signaling pathway	6/34	363/18670	4.40e-05	0.018	0.014
CC	GO:0070382	exocytic vesicle	5/38	207/19717	4.60e-05	0.005	0.005
CC	GO:0098793	presynapse	6/38	491/19717	3.24e-04	0.018	0.016
CC	GO:0008021	synaptic vesicle	4/38	191/19717	4.87e-04	0.018	0.016
MF	GO:0015081	sodium ion transmembrane transporter activity	4/32	149/17697	1.44e-04	0.007	0.004
MF	GO:0099516	ion antiporter activity	3/32	58/17697	1.55e-04	0.007	0.004
MF	GO:0015386	potassium: proton antiporter activity	2/32	11/17697	1.72e-04	0.007	0.004
KEGG	hsa04310	Wnt signaling pathway	6/20	160/8076	1.70e-06	8.82e-05	8.03e-05

### *In Vitro* Validation of DIRAS2 Function in SKCM

**Figure 4:**
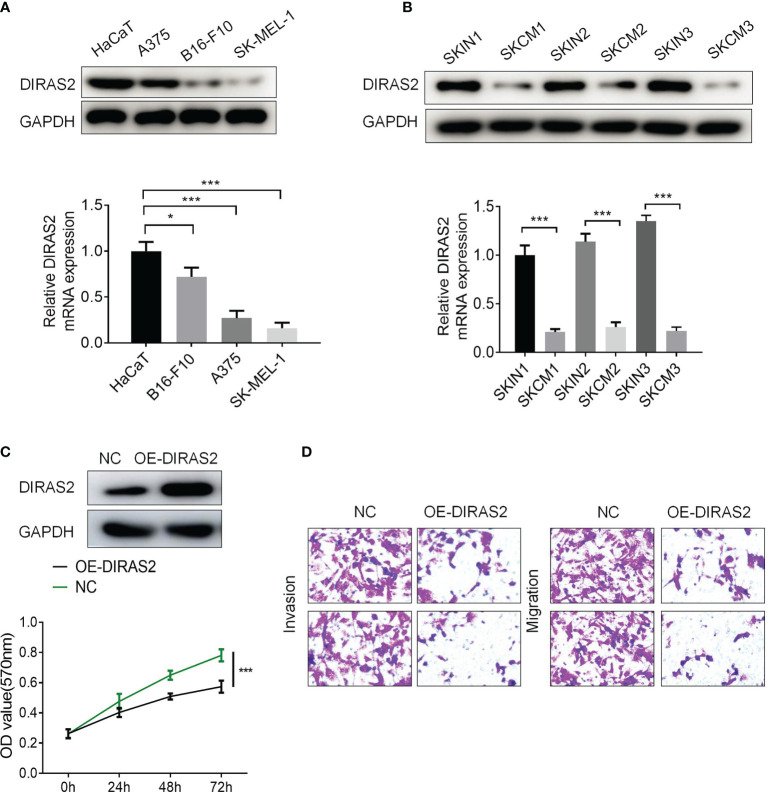
DIRAS2 overexpression depresses the proliferation, invasion, and migration ability of SKCM. **(A)** Western blot and qRT-PCR showed the expression of DIRAS2 in HaCaT and SKCM cells lines. **(B)** DIRAS2 expression was detected in normal skin and SKCM using Western blot and qRT-PCR. **(C)** Transfection efficiency was verified after transfection of the DIRAS2 overexpression plasmid. SKCM cell proliferation potential was evaluated using CCK-8 assays at 0, 24, 48, and 72 h post-transfection. **(D)** Transwell assays were used to detect SKCM invasion and migration after transfection of the DIRAS2 overexpression plasmid. Representative experiments were shown. *p < 0.05; ***p < 0.001.

To explore the expression of DIRAS2 in SKCM cell lines, it was found through experiments that DIRAS2 was lowly expressed in SKCM cell lines compared with HaCaT normal cells, while DIRAS2 was expressed lower in SK-MEL-1 than in A375 and B16-F10 cell lines (**Figure 4A**). DIRAS2 expression was lower in SKCM tissue than in normal skin tissue, according to the results of the western blot and qRT-PCR analysis (**Figure 4B**). Transfecting SK-MEL-1 cells with a DIRAS2 overexpression plasmid produced significant transfection efficiency (**Figure 4C**). In addition, DIRAS2 overexpression depressed the proliferation, invasion, and metastatic ability of SK-MEL-1 cells (**Figures 4C, D**).

### Downregulation of DIRAS2 Promotes SKCM Cell Proliferation, Invasion, and Migration

**Figure 5:**
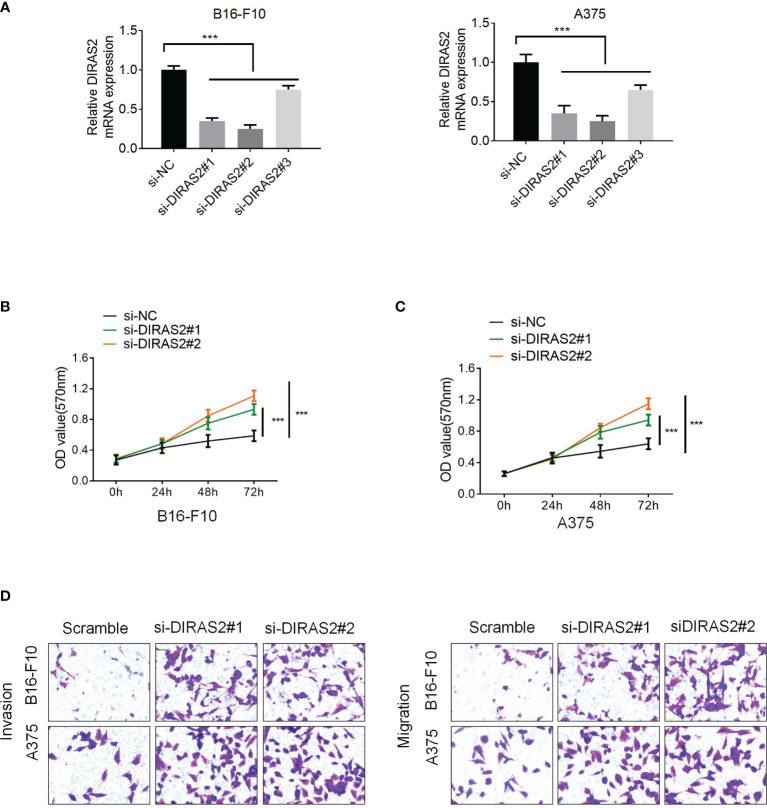
Downregulation of DIRAS2 promotes SKCM cell proliferation, invasion, and migration. **(A)** After transfection of DIRAS2 or a negative control siRNA, transfection efficiency was determined. **(B)** After the DIRAS2 knockdown, the number of SKCM cell colonies increased. **(C)** SKCM cell proliferation potential was evaluated using CCK-8 assays at 0, 24, 48, and 72 h post-transfection. **(D)** Transwell assays were used to detect SKCM invasion and migration. Representative experiments were shown. Data are represented as the mean ± SD of three independent experiments. n = 3, ***p < 0.001.

Moreover, three siRNA sequences were designed that interfered with DIRAS2 expression: si-DIRAS2#1, si-DIRAS2#2, and si-DIRAS2#3. Then, transfected into A375 and B16-F10 cells, and the results exhibited that after DIRAS2 knockdown, the relative expression levels of tumor cells in the cell lines dwindled (**Figure 5A**, P < 0.001). The knockdown efficiency of si-DIRAS2#3 in the two groups of cell lines was not as high as those in si-DIRAS2#1 and si-DIRAS2#2. As a result, for the following investigation, two interference sequences (si-DIRAS2#1 and si-DIRAS2#2) were selected. Using a cell clone formation experiment, the effect of DIRAS2 on SKCM cell proliferation was confirmed. Results exhibited thatreducingDIRAS2 expression could significantly enhance the colony-forming potential of SKCM cells (**Figure 5B**). Additionally, the effects of DIRAS2 on cell proliferation were measured using the CCK-8 method. The proliferation of tumor cells was notably enhanced after knocking down DIRAS2 (**Figure 5C**). Furthermore, Transwell assays revealed that DIRAS2-knockdown SKCM cells had significantly increased invasion and migration potential (**Figure 5D**).

### Expression of Wnt/β-Catenin Related Protein in DIRAS2 Silenced Cells

**Figure 6:**
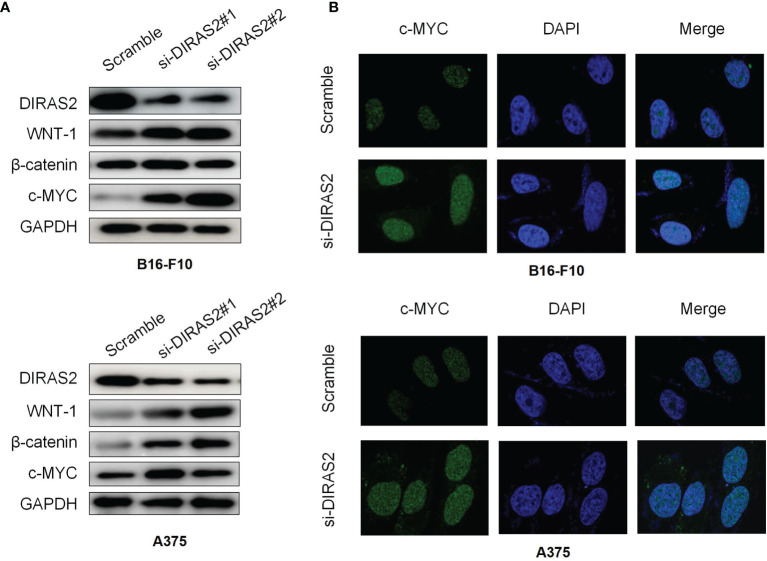
Expression of Wnt/β-catenin related protein in DIRAS2 silenced cells **(A)** DIRAS2 expression was down-regulated. Wnt-1 and β-catenin protein levels were measured in B16-F10 and A375 cells. **(B)** Cellular immunofluorescence experiment to detect the expression of Wnt signaling pathway-related protein c-myc in SKCM cells. Data are represented as the mean meant to detect the expression of the Wnt signaling pathway.

β-catenin is associated with RAS in tumorigenesis and that activated RAS can be activated by promoting β-catenin tyrosine phosphorylation, resulting in a cross-reaction between RAS/MAPK and Wnt/β-catenin signaling pathways. To study the relationship between DIRAS2 and Wnt/β-catenin signaling pathway in SKCM, a western blot was adopted to detect the important factors of the Wnt/β-catenin pathway, Wnt-1, β-catenin, and c-MYC. The results demonstrate that the expression levels of Wnt1 and β-catenin in the siRNA-DIRAS2 group were significantly higher than those in the NC group in B16-F10 and A375 cells. In B16-F10 cells and A375 cells, as the expression of DIRAS2 was down-regulated, the Wnt/β-catenin signaling pathway was activated (**Figure 6A**). The cellular immunofluorescence investigation, as shown in the figure, revealed that after silencing DIRAS2, the nucleus fluorescence brightness of c-myc in SKCM cells increased, and its content was up-regulated compared to the NC group (**Figure 6B**). This result indicates that silencing DIRAS2 can activate the signal transduction of the Wnt/β-catenin signaling pathway.

### Knocking Down of DIRAS2 Promoted Melanoma Growth and Metastasis *In Vivo*

**Figure 7:**
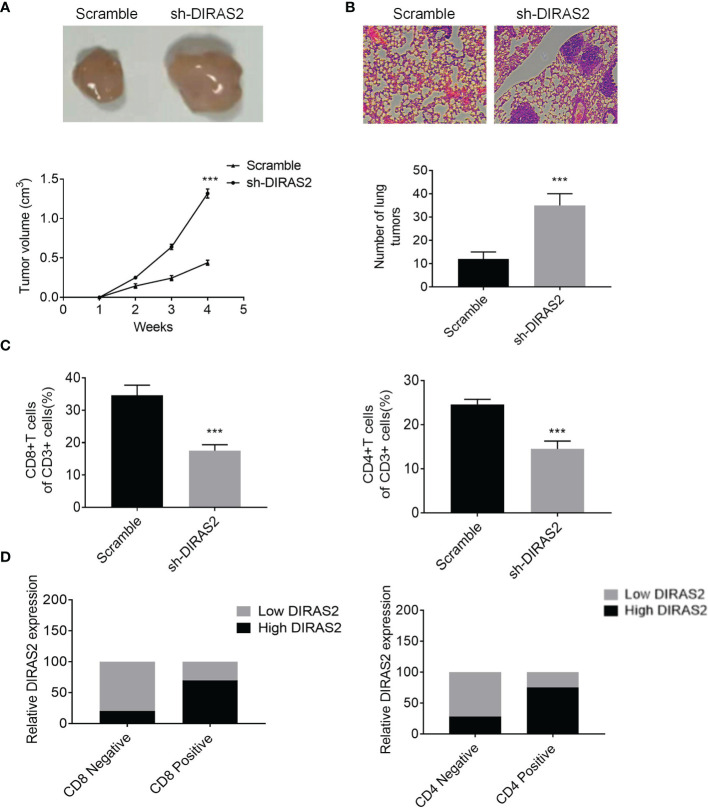
DIRAS2 inhibits melanoma growth and metastasis *in vivo*. **(A)** Representation of tumor bodies with tumor growth curves (n=5). **(B)** HE staining of lung tissue with statistics on lung tumor number. **(C)** The percentages of CD8+ and CD4+ T cells in tumor tissues with CD3+ cells. **(D)** Expression levels of DIRAS2 in melanoma tissues in CD4 or CD8 negative or positive specimens (n=20). Data are represented as the mean ± SD of three independent experiments. ***P < 0.001.

As shown in **Figure 7A**, the size of tumors in the DIRAS2 down-regulated group was significantly larger than that of the control group, and the growth rate of tumors in the down-regulated DIRAS2 group was significantly faster than the control group. Lung tissues were obtained from nude mice and stained with H&E to confirm these findings *in vivo*. The findings revealed that the DIRAS2 down-regulated group’s alveolar tissues included denser tumor cell islands than the control group (**Figure 7B**). This indicates that down-regulated of DIRAS2 promoted lung metastasis.

Flow cytometry analysis of the immune landscape of transplanted tumors revealed that suppressing DIRAS2 significantly diminished the proportion of CD4+ and CD8+ T cells (**Figure 7C**). In addition, to verify the relationship between DIRAS2 and immune cells *in vivo*, we used an IHC assay to detect the positive rate of CD4+ T and CD8+ T cells in the tissues of 20 melanoma patients. Statistical analysis showed that the expression of CD4+ T and CD8+ T was significantly higher in the high DIRAS2 expression group, while the expression of CD4+ T and CD8+ T cells was significantly lower in the low DIRAS2 expression group (**Figure 7D**). This result suggests that DIRAS2 may affect tumor development through CD8+ and CD4+ T cells.

## Discussion

DIRAS2 is a subgroup of the RAS family that shares 30-40% of its DNA with other members. The DIRAS family in humans consists of three functional genes, *DIRAS1, DIRAS2*, and *DIRAS3*, which are distributed across multiple chromosomes and tissues (12, 18). It is well-known that the Ras superfamily is oncogenic, however, despite a high degree of homology with members of the RAS superfamily, each individual DIRAS plays a very different role in regulating cellular function and cancer development. Several studies indicate that members of the DIRAS family may function as tumor suppressors in a variety of cancers (13, 19–22). The role of DIRAS1 and DIRAS3 in tumors has been widely investigated (20, 23–27), but research on DIRAS2 has mainly focused on ADHD (28–31), and its role in tumors has not been extensively studied. Furthermore, only two investigations have suggested its role in tumor progression, with conflicting conclusions about tumor inhibition or tumor promotion. Sutton et al. demonstrated that DIRAS2 reduced ovarian cancer growth *in vitro* when coupled with DIRAS1 (13). On the contrary, however, Rao et al. displayed that DIRAS2 could potentially function as an oncogene in clear cell renal cell carcinoma (14). It was found here that DIRAS2 expression is significantly different in SKCM tumor tissues and paracancerous normal tissues, and is low in SKCM tumor tissues, using the GEPIA online database; the same result was obtained in GEO data set verification. DIRAS2 expression is associated with survival/prognosis in SKCM patients. To further study the expression mechanism and function of DIRAS2 in SKCM, we downloaded the dataset from TCGA. DIRAS2 expression and tumor stage were found to be independent prognostic factors in SKCM patients in a multivariate study. As a result, DIRAS2 might become a potential prognostic biomarker for SKCM.

Further study revealed that the expression of DIRAS2 in SKCM was correlated with the level of immune invasion. A CIBERSORT analysis demonstrated that the expression of DIRAS2 was positively correlated with the infiltration levels of aDC, B cells, CD8 T cells, cytotoxic cells, eosinophils, iDC, macrophages, neutrophils, NK CD56dim cells, T cells, T helper cells, Tcm, Tem, TFH, Th1 cells, Th2 cells and TReg in SKCM. Further analysis using TIMER revealed that DIRAS2 expression in SKCM was distinctly correlated with B cells, CD4+ T cells, and CD8+ T cells, all of which positively correlated with SKCM patient survival rates. These findings suggest that DIRAS2 has a tumor suppressor role in SKCM, which may be related to immune infiltrating cells in the tumor. *In vitro* experiments were conducted to confirm that DIRAS2 indeed has anti-cancer functions in SKCM cells. This tumor-suppressive property is consistent with the outcomes of Sutton et al.’s study (13), but runs counter to the results of Rao H et al.’s study (14).

In recent years, growing evidence suggests that tumor microenvironments (TME) play an important role between stromal cells and tumor-infiltrating immune cells (TICs) (32). TICs have been found as a promising TME index for assessing therapeutic efficacy in several investigations (33). Here, it was found significant infiltration of CD8+ T cells, CD4+ T cells, B cells, dendritic cells (DC), macrophages, and neutrophils in SKCM. Cumulative survival was associated with CD8+ T cells, B cells, dendritic cells (DC), and neutrophils (34). CD8 + T cells play a critical role in the adaptive immune response to cancer (35). Cytotoxic T lymphocytes (CTL) are activated CD8 + T cells that can directly detect and kill malignant and contaminated cells (36, 37). By promoting cytotoxicity to tumor cells, generating tumor-specific antibodies, and as APC, B cells can impede tumor development, especially when DC may be absent or impaired (38, 39). Macrophages also have dual properties and can be categorized into two types: pro-inflammatory or anti-tumor M1-type and anti-inflammatory or pro-tumor M2-type macrophages (40). Targeting macrophage anti-tumor activities is believed to be a potential strategy of melanoma suppression. It was found significant macrophage infiltration in SKCM; however, this infiltration was not linked with cumulative survival. Therefore, we hypothesized that M2-type macrophages would be essential. Of course, the specific immune mechanism still needs clinical and experimental study.

Our results suggest a correlation between DIRAS2 and the prognosis and immune infiltration of SKCM. Statistical analysis revealed that the expression of CD4+ T and CD8+ T cells was significantly higher in the high DIRAS2 expression group; whereas it was significantly lower in the low DIRAS2 expression group. This result suggests that DIRAS2 may affect tumor development through CD8+ and CD4+ T cells. In other words, DIRAS2 has a significant effect on SKCM immune infiltration, while the positive effect of DIRAS2 on SKCM may be related to the high density of these TICs, and DIRAS2 may play an important role in the regulation of the tumor immune microenvironment in SKCM patients.

Then, the exact mechanism of DIRAS2 on the growth, proliferation, and migration of SKCM cells, and the mechanisms responsible for these changes stimulate our interest in further research. The Wnt signaling pathway is a key signaling pathway involved in tumorigenesis. It was confirmed that the key molecules and downstream effectors of the Wnt pathway in our study. The Wnt signaling pathway is very conservative in evolution and plays an important role in tissue regeneration and maintaining the internal environment’s stability (41–43). β-catenin is a key transcriptional co-activator in the typical Wnt signaling pathway and an intracellular signal converter of the Wnt signaling pathway, which can tightly regulate cell adhesion and play a key role in tumorigenesis (44). The binding of the Wnt ligand and the corresponding receptor can stimulate the Wnt/β-catenin signaling pathway (45). Wnt-1 is the first member of the Wnt family, which can promote cell proliferation and migration, prolong the survival time of cancer cells, and promote cancer progression (46). C-myc is a proto-oncogene, which can encode nucleophosphoprotein. Its expression is up-regulated in a variety of malignant tumors as a key effector molecule downstream of the Wnt/β-Catenin signaling pathway (47–49). The DIRAS2 gene was silenced to down-regulate the expression level of SKCM cells, and then detect the expression of important components in the Wnt/β-Catenin pathway. The results indicated that when DIRAS2 expression was reduced, the expression levels of Wnt-1 and β-catenin protein increased significantly, as did the expression of c-myc. Based on this judgment, DIRAS2 may negatively regulate the Wnt/β-Catenin signaling pathway, leading to down-regulation of the expression of Wnt signaling pathway-related proteins, and participating in the inhibition of the formation and development of SKCM.

Based on the findings, including the results of *in vitro* tests, it was observed that DIRAS2 is a tumor suppressor gene for SKCM, with the Wnt/β-Catenin signaling pathway being the negatively regulated target. DIRAS2 can be a molecular marker for the prognosis of SKCM, and thus may become a therapeutic target. Because of the possible benefits of SKCM treatment, more research on this topic is strongly recommended.

## Data Availability Statement

The original contributions presented in the study are included in the article/**Supplementary Material**. Further inquiries can be directed to the corresponding author.

## Ethics Statement

This study was conducted by the Institutional Review Committee of the First Hospital of Shanxi Medical University as well as Hengyang Medical School and was conducted in accordance with the Helsinki Declaration. The patients/participants provided their written informed consent to participate in this study. The NOD/SCID mice were employed to establish an animal model of melanoma metastasis. Briefly, 5x105 A375 cells were injected into the mice into the tail vein once a day for 3 days. The mice were finally euthanized and H&E stained was used to observe the lung metastases. All procedures were approved by the Animal Care and Use Committee of the First Hospital of Shanxi Medical University as well as Hengyang Medical School.

## Author Contributions

WX and HL designed this study. WX and HH performed the analyses. WX wrote the manuscript. HL and HH reviewed the data and manuscript. HZ conducted the article revision. All authors contributed to the article and approved the submitted version.

## Conflict of Interest

The authors declare that the research was conducted in the absence of any commercial or financial relationships that could be construed as a potential conflict of interest.

## Publisher’s Note

All claims expressed in this article are solely those of the authors and do not necessarily represent those of their affiliated organizations, or those of the publisher, the editors and the reviewers. Any product that may be evaluated in this article, or claim that may be made by its manufacturer, is not guaranteed or endorsed by the publisher.
